# From digital to physical model: the use of 3D-printed models in wound ballistic reconstruction

**DOI:** 10.1007/s00414-025-03475-5

**Published:** 2025-03-29

**Authors:** Fabiano Riva, Daan Wintermans, Stefan Schaufelbühl, Nadine Fuchs, Wim Kerkhoff

**Affiliations:** 1 University Center of Legal Medicine Lausanne, University Hospital Lausanne, Lausanne, Switzerland; 2 Ecole Des Sciences Criminelles, Université de Lausanne, Lausanne, Switzerland; 3 Netherlands Forensic Institute (NFI), PO Box 24044, 2490 AA The Hague, The Netherlands; 4 Forensic Institute of St. Gallen, Cantonal Police St. Gallen, St. Gallen, Switzerland

**Keywords:** Wound ballistics, Simulants, Head models, 3D printed bones

## Abstract

Synthetic models (also called “surrogates”) simulating human tissues are widely used in wound ballistics. Although there are a large number of commercial models showing interesting properties, these are limited to generic shapes. The result of the interaction between the projectile and the target varies based on several parameters; therefore, using a case-specific, custom-shaped synthetic model would enhance the accuracy of the findings. For this purpose, the authors created, based on Post-Mortem Computed Tomography (PMCT) measurements, case specific 3D-printed synthetic models. The first ballistic tests were performed on simple plates printed with different materials and compared against polyurethan Synbone® products in order to select the most suited materials for synthetic head models. Further tests were realised on head models printed with PLA (polylactic acid), PETG (polyethylene terephthalate glycol-modified) and TPU (thermoplastic polyurethane) polymers as well as on two head models composed of powder and resin. The bullet’s behaviour, its deformation, the wound channel and other qualitative aspects were directly compared to the findings of the real case reported in Riva et al in Int J Legal Med 135:2567–2579, 2021, as well as to the “open shape” head model created by Riva et al in Forensic Sci Int 294:150-159, 2019. Finally, although the results of this study did not completely fulfil the requirements to simulate human bones, its concept in reproducing case specific head models with easily available 3D printing materials, is very promising.

## Introduction

### Wound ballistic reconstructions

The properties of biological tissues under high-velocity loading are still largely unknown. Therefore, acquiring knowledge on bullet behaviour in human tissues (soft tissue, bone, skin, etc.) still depends on physical experimentation with so-called simulants or surrogates. Pertaining to bone, Synbone® products are at present the most commonly used simulants. These simulants can, for example, be used to reproduce characteristics of wounds observed after a shooting incident, in order to verify or falsify relevant hypotheses. Bone simulants were applied in a forensic context by [1–8]. Other research [9–11] compared bullet fractures that occurred in Synbone® “generic spheres” (hollow spheres with a diameter of 19 cm) to cranial fractures observed after fatal incidents with humans [3, 12]. Kneubuehl et al. [13] and Henwood et al. [3] compared various experimental results from Synbone® products to experimental results from bovine and porcine bone. The results of these studies varied, in the sense that some results with Synbone® were similar to human and animal bone and some were not.

The need to select a simulant to replicate a specific situation with a specific victim is partially met by the fact that Synbone® generic spheres can be acquired with a wall thickness of 5, 6 and 7 mm. However helpful, this range cannot cover the wide variety of cranial bone thickness observed in humans. De Boer et.al [14] observed a wide variety of cranial thicknesses in a dataset of 1097 human samples, with several measurements below 4 mm and above 10 mm. Rowbotam [15] measured cranial thickness at 20 points across the frontal, parietal, temporal and occipital bones on post-mortem CT-scans of 604 individuals. At the lower end, several values below 2 mm were measured at the temporal squama. At the higher end of the measurements, extremes exceeding 14 mm were measured with several individuals on both the parietal and occipital bones. Mean overall thickness of the occipital protuberance was around 15 mm, with an extreme measurement exceeding 30 mm. This diversity in the human population illustrates the need for custom made simulants for optimised wound ballistic experimentation. A step in this direction was taken e.g. by Mahoney et al. [16]. The authors of that study used anatomically correct polyurethane skull simulants instead of spheres. Though resembling an actual skull, these simulants were still generic in the sense that they were derived from scanned bodies unrelated to the victims of the case that the authors studied. Given the observed variation in cranial thickness, it is impossible to produce an anatomically correct skull simulant that suffices in every single case. Riva et al. [2] produced “open shape” head models for ballistics tests with customised sizes by assembling Synbone® plates in order to reach the skull thickness based on Post-Mortem Computer Tomography (PMCT) measurements. This type of simulant is a step forward in customisation, but the resulting head model still represents a rough approximation. The need for customised skull simulants in forensic casework, coupled with the possibilities offered by the emerging 3D printing technologies, triggered the current study.

### 3D printing

The fundamental principles of additive manufacturing were first established in the 1960s, with the initial patents emerging in the 1980s [17, 18]. The technology, now more commonly referred to as 3D printing, employs a process of successive deposition of material in order to build objects layer by layer. Following the commercialisation of 3D printers in the mid-2000s and after significant advancements, this technology is now widely used in both the industrial and private sectors. At the time of writing, seven printing processes are available. Of these, Material Extrusion (MEX) and Vat Photopolymerization (VPP) are the most commonly used outside of industry [19–22]. Meanwhile, 3D printing is also used for various purposes in forensic science and legal medicine. In these fields, 3D-printed replicas of evidence or related objects serve as aids for presentation and/or reconstruction purposes in caseworks and in court [23–25]. Such physical replicas often provide additional illustrative information, which may be challenging to discern or even be absent in photographic materials. Two studies assessed the quality and accuracy of 3D printed teeth and human skulls and concluded that 3D printing technology is indeed capable of accurately reproducing such objects [26, 27]. Another study assessed the surface quality of 3D-printed bones and concluded that while gross features were accurately reproduced, there were limitations when it came to observing finer features (e.g. bone texture) on the reproduced bones [28]. Finally, the authors of these studies emphasise that the methods used during the acquisition process (3D scanning of the object) and the replication by 3D printing have a strong influence on the final quality and accuracy of these objects. Choosing appropriate methods in these areas is therefore of paramount importance.

### Scope of the current study

The purpose of the current study was to find and optimize a method for producing a 3D-printed bone simulant for wound ballistics tests, which can be used to reproduce the wound characteristics observed in shooting incidents. The steps of this study can be summarised as follows:

1. create a 3D model from the PMCT data of a well-documented fatal headshot;
2. create a skull simulant from that model using 3D printing technology;
3. integrate the resulting customised 3D printed simulant in a whole head model;
4. submit the head model to ballistics tests, replicating the circumstances of the case and compare the results of the ballistics tests to the case information.

In a preliminary stage, several printable candidate materials were selected and subjected to ballistic loading. The results were compared to those of Synbone® samples.

## Materials and methods

### Preliminary tests

Based on a preliminary study conducted by the authors, PLA, PETG, and TPU were identified as the most promising, cost-effective, and widely available printable polymers for this study. All three products were acquired from Extruder® [29]. ®The density of the used TPU (Flex hard, 1.20 g/cm^3^) was a little lower. PLA and PETG are widely available and easy to use 3D printing filaments. Working with TPU requires slightly more user experience, but remains relatively easy to print, especially with modern 3D printers that offer advanced user support and settings optimization.

**Fig. 1:**
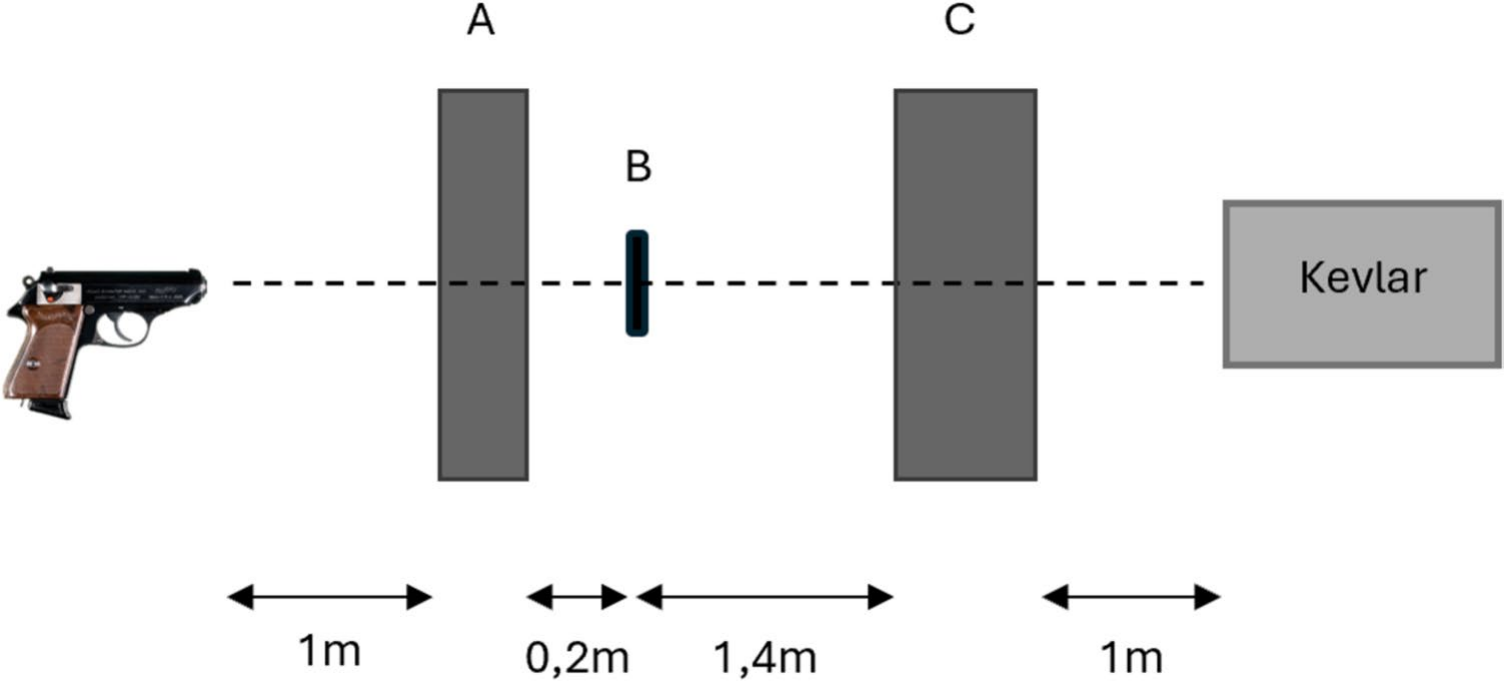
Set-up used during preliminary tests. The velocity of the bullet was measured before the plate with the first 0.5 m detector (**A**) and behind the plate with the second 1.0 m detector (**C**)

In a series of preliminary tests, the energy loss of projectiles fired through these materials was measured and compared to that of Synbone®. Synbone® was chosen as a reference, because previous studies [1–3, 7, 9, 10, 12, 16] indicated that the properties of these products are close to that of human bone under ballistic loading. PLA, PETG and TPU plates were 3D-printed, some with two infill densities (70% and 100%). Because the literature suggests that bullets deform on the cortical bone [30], some plates have been printed with a filling percentage less than 100%; the remaining space has been filled with epoxy resin (Crystal® Clear Casting epoxy resin). By doing so, it was hoped to create a harder and denser plate, which could simulate the cortical bone behaviour. The 80 × 80x6 mm printed sample plates and Synbone® reference plates, cut to equal size, were mounted in a fixture. The plates were shot with 0.22 Long Rifle bullets, fired from a rifle (mean velocity 363 ± 6 m/s). Bullet velocity before and behind the plates was measured with two Drello® (Germany) velocity detectors with respectively 0.5 m (detector A in Fig. 1) and 1.0 m (detector C in Fig. 1) basis length. Energy loss was calculated with the recorded velocities and bullet mass of 2.55 g. This first test series revealed that the PLA, PETG and TPU samples printed with 100% infill densities were the most promising, in the sense that energy loss came closest to that of the Synbone® plates. This test was also realised with 7.65 mm Browning FMJ bullets (4.60 g), fired from a pistol (mean velocity 270 ± 6 m/s). See Fig. 1 for a sketch of the set-up.

The test performed with 7.65 mm Browning FMJ bullets confirmed that the properties of the samples (PLA, PETG and TPU) printed with 100% infill density were close in terms of energy loss to those of Synbone® under ballistic loading. According to the obtained results, filling less than 100% and the external application of epoxy resins have not been considered for further tests. PLA, PETG and TPU, printed with 100% infill density were eventually used in the head model tests. Because no results for energy loss under ballistic loading of 3D printed materials could be found in the literature, the results of all preliminary tests will be presented in the Section "Preliminary tests".

### Shooting tests on head models

#### Fatal shooting

**Fig. 2:**
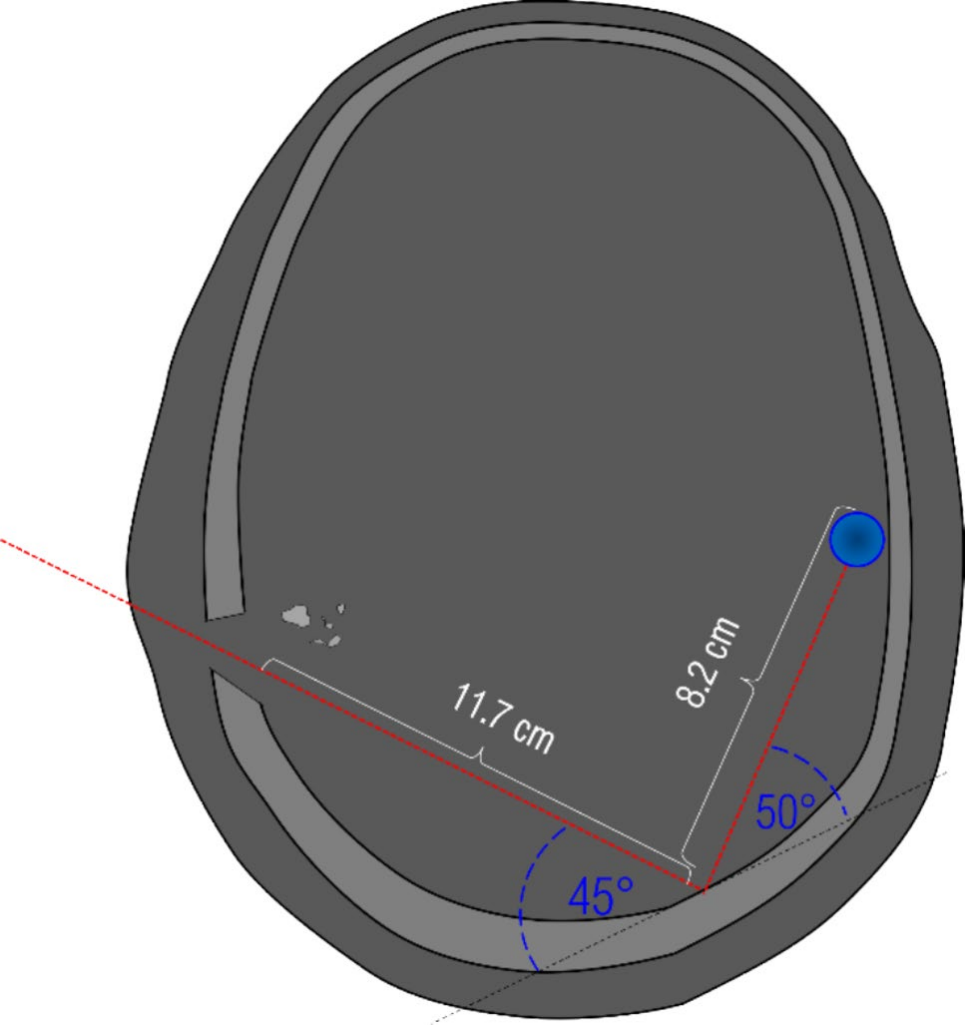
Illustration of the projectile’s path and its end position in the victim’s head

The shot concerned a direct (no intermediate target) headshot with a 6.35 mm Browning calibre pistol at close range. The FMJ projectile was retained in the victim’s cranial vault. According to the findings reported by the forensic pathologists and the radiologists, the bullet entered the skull in the right parieto-temporal region. An acute linear intra-parenchymal haemorrhage in the right parietal and temporal lobe extending to the left occipital lobe passing through the posterior horn of the right lateral ventricle, the right venous sinus and the middle line (approx. 12 cm of length) was observed and associated to the projectile penetration. This linear haemorrhage extended further from the left occipital lobe to the left temporal lobe (approx. 8 cm of length) caused after a presumed projectile ricochet against the inner wall of the occipital bone. Figure 2 shows a drawing of the CT findings illustrating the projectile path in the victim’s head.

The projectile was extracted during the autopsy. Its surface showed a flat diagonal deformation starting from the top of the projectile and a less pronounced flat deformation on the opposite side of the projectile. The projectile was also slightly flattened at the base.

#### From PMCT data to the G-code

The AW Server software was used to analyse DICOM data from the PMCT and to measure the different sections of the projectile’s path through the different tissues: skin, subcutaneous layer, bone and brain. The same software was used to isolate, through a segmentation process, the bone structure of the skull from the other tissues (brain and skin) based on its density. A 3D model of the skull was then exported in an STL format.

**Fig. 3:**
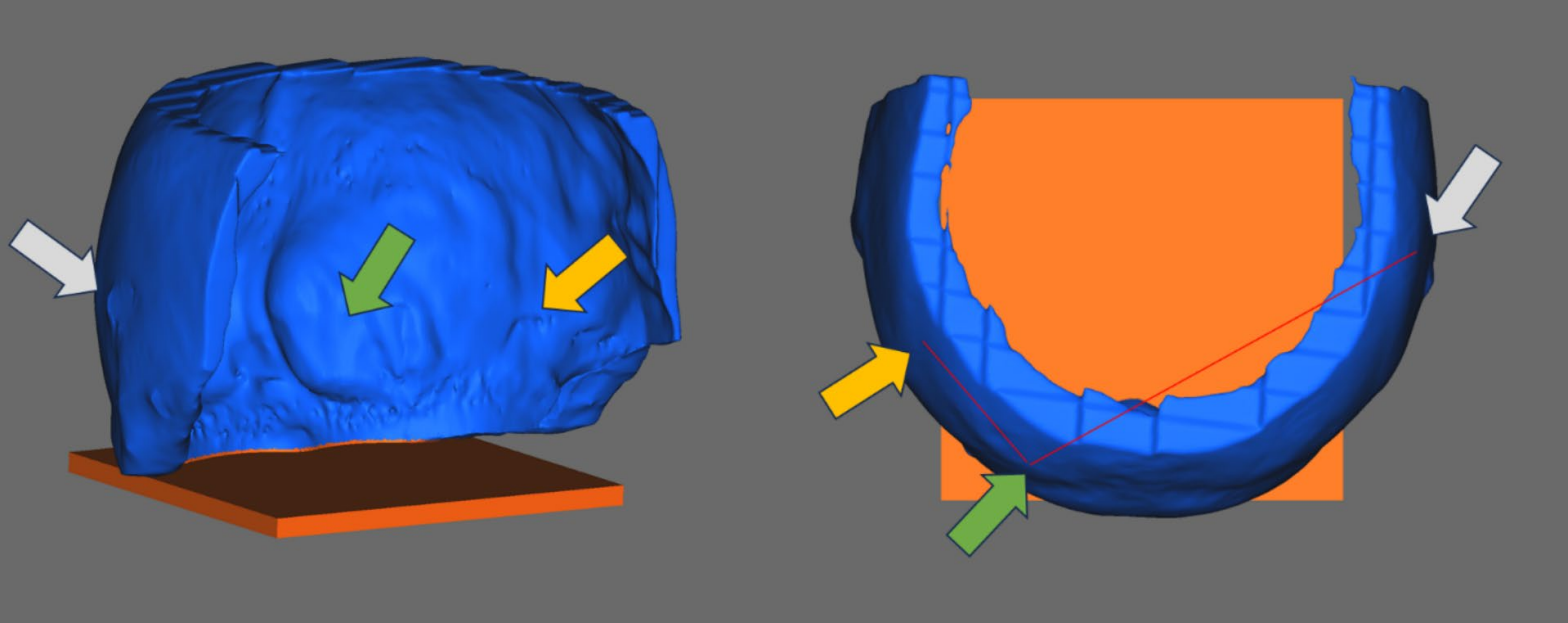
Representation of the resulting 3D model (3D view and from above)

From the resulting 3D skull model, the upper and frontal part of the skull were segmented to select the region of interest from a ballistics point of view. The entry hole of the projectile was filled. Micro holes (lack of data) and noise were eliminated by applying a smoothing function. These operations were performed with GOM Inspect software. Finally, a squared base (orange part in Fig. 3) was added to the 3D model (blue part in Fig. 3) in order to facilitate the further shooting setup (Section "Shooting tests setup"). Figure 3 shows an image of the resulting 3D model. The filled-up entry hole, the ricochet emplacement in the occipital region as well as the end of the wound channel caused by the projectile are respectively highlighted in the Fig. 3 by the grey, the green and the yellow arrow.

#### 3D printing parameters and materials

As mentioned with the preliminary tests, the properties of the PLA, PETG and TPU samples printed with 100% infill density were the most promising as bone simulants. For the shooting tests on the head models, the authors had the opportunity to print some additional samples, using industrial 3D printers based on the Binder Jetting (BJT) and Material Jetting (MJT) processes. In total, eleven 3D-printed skull models were created for this test. The printing parameters of the samples and the number of samples are listed in Table 1.

**Table 1:** Tested materials and printing parameters

3D printer	Material	Printing parameters		# of prints
		Parameter	Value	
Prusa i3 MK3s + (MEX process)	Extrudr NX2 PLA	Layer height: Infill density: Infill pattern: Perimeter count: Nozzle T°: Build plate T°:	0.30 mm 100% Rectilinear 3 215 °C 60 °C	3
Prusa i3 MK3s + (MEX process)	Extrudr PETG	Layer height: Infill density: Infill pattern: Perimeter count: Nozzle T°: Build plate T°:	0.30 mm 100% Rectilinear 3 220 °C 75 °C	3
Prusa i3 MK3s + (MEX process)	Extrudr Flex Hard (TPU)	Layer height: Infill density: Infill pattern: Perimeter count: Nozzle T°: Build plate T°:	0.20 mm 100% Rectilinear 3 240 °C 70 °C	3
3D-Systems ZPrinter 150 (BJT)	zp151 powder with zb63 Clear Binder	Layer height: Infill density:	0.102 mm 100%	1
3D-Systems Projet 3510 SD (MJT)	VisiJet M3 Crystal	Layer height: Infill density:	0.032 mm 100%	1

For the MEX and MJT samples, post-processing was required to achieve the final product. Support structures used during printing were removed, and the parts were manually finished by sanding and eliminating any remaining artifacts. Furthermore, the MJT-printed parts underwent UV curing to ensure complete material hardening. For the BJT sample, post-processing involved depowdering to remove any unfused powder from the printed part.

#### Integration of the skulls into the head models

With the exception of the 3D-printed skull, the head model used in this study has several similarities with the “open shape” model used by Riva et al. [1]. It is composed of (a) a 1 mm thick layer of leather to simulate the skin; (b) a layer of gelatine to simulate the subcutaneous tissues, (c) 3D-printed material to simulate the skull and gelatine to simulate the brain. The leather skin simulant (cowhide, semi-finished chrome tanned upholstery “crust”) was chosen following Jussilla et al. [31]. The gelatine that was used to simulate the subcutaneous layer and the brain was prepared with 10% (by weight) gelatine (Type 3, 250 Bloom number, Gelita, Eberbach, Germany) and cooled to 4 °C. It was prepared and calibrated in accordance with the guidelines provided by Jussilla et al. [32]. The dimensions of the head model and of each tissue layer considered in relation to the wound track correspond to the measurements performed on the PMCT images (Fig. 2) of the victim [1].

**Fig. 4:**
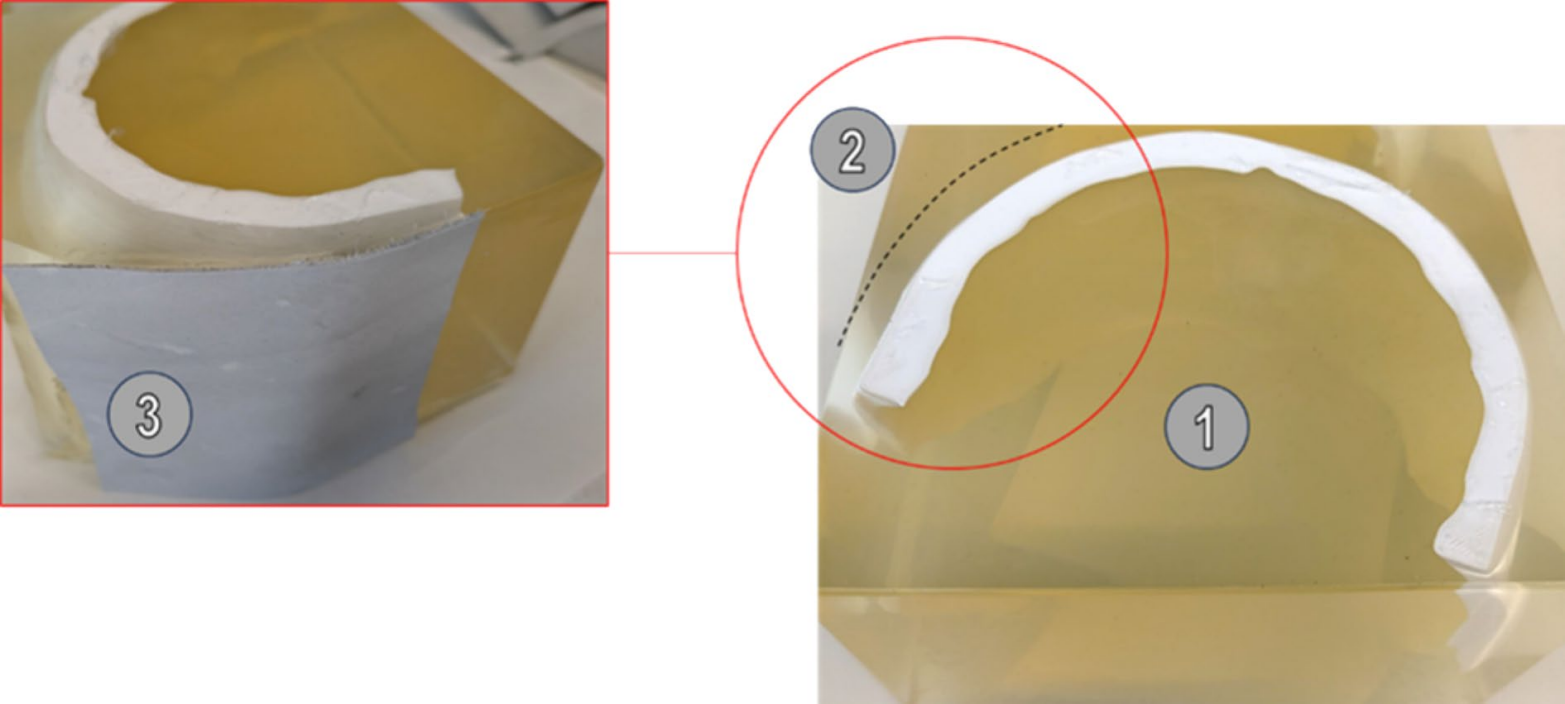
Preparation of the head models

The models were prepared as follows: the 3D-printed bone simulants were introduced into a mould and the liquid gelatine was poured into it. The models were then placed for 48 h into a refrigerator at approximately 4 °C. Once the gelatine had solidified, (#1 in Fig. 4), it was cut in order to obtain the suitable subcutaneous layer thickness (#2 in Fig. 4 – black dotted line) and the leather was applied to the front of the model (corresponding to the right temporal region) by heating the gelatine with a hairdryer (#3 in Fig. 4). Finally, the location of the entry wound was marked on the skin simulant and the models were placed again into the refrigerator at 3–4 °C until the shooting tests.

During the gelatine preparation, a portion was set aside for verification prior to proceeding with the ballistics tests. The gelatine verification was performed by shooting two BB’s at two different velocities as recommended by Jussila et al. [32].

#### Shooting tests setup

The pistol used for the ballistics tests on the 3D-printed head models was an Astra CUB semi-automatic pistol calibre 6.35 mm Browning (0.25 Auto) with a 5 cm (2″) barrel and the ammunition was a Geco cartridge equipped with a brass Full Metal Jacket (FMJ) projectile weighting 3.2 g (50 grains). Both the pistol and the ammunition were the same as used in Riva et al. [1].

A series of 18 shots were performed in order to assess the precision of the pistol and, at the same time, to record the velocity of the projectiles. In order to do so, the pistol was fixed in a ransom rest at 1 m distance from a cardboard target. A velocity detector with a 0.5 m basis (Drello®, Germany) was placed with its centre in the middle of the trajectory at 0.5 m from the pistol and 0.5 m from the target. For each shot, the velocity has been recorded and the distance between the point of aim and point of impact was noted. The velocity of the projectiles was 228 ± 3 m/s. However, the precision of the pistol has been deemed insufficient to reliably hit the head model precisely at the desired position of the entry at a shooting distance of 1 m. For this reason and considering the consistent reproducibility of the muzzle velocity, the shooting tests on the head models were performed at a shorter shooting distance of approximately 0.2 m, removing the velocity detector between the weapon and the target. The velocity increase at the impact caused by the shorter distance has been calculated and has been judged not significant (less than 1 m/s).

**Fig. 5:**
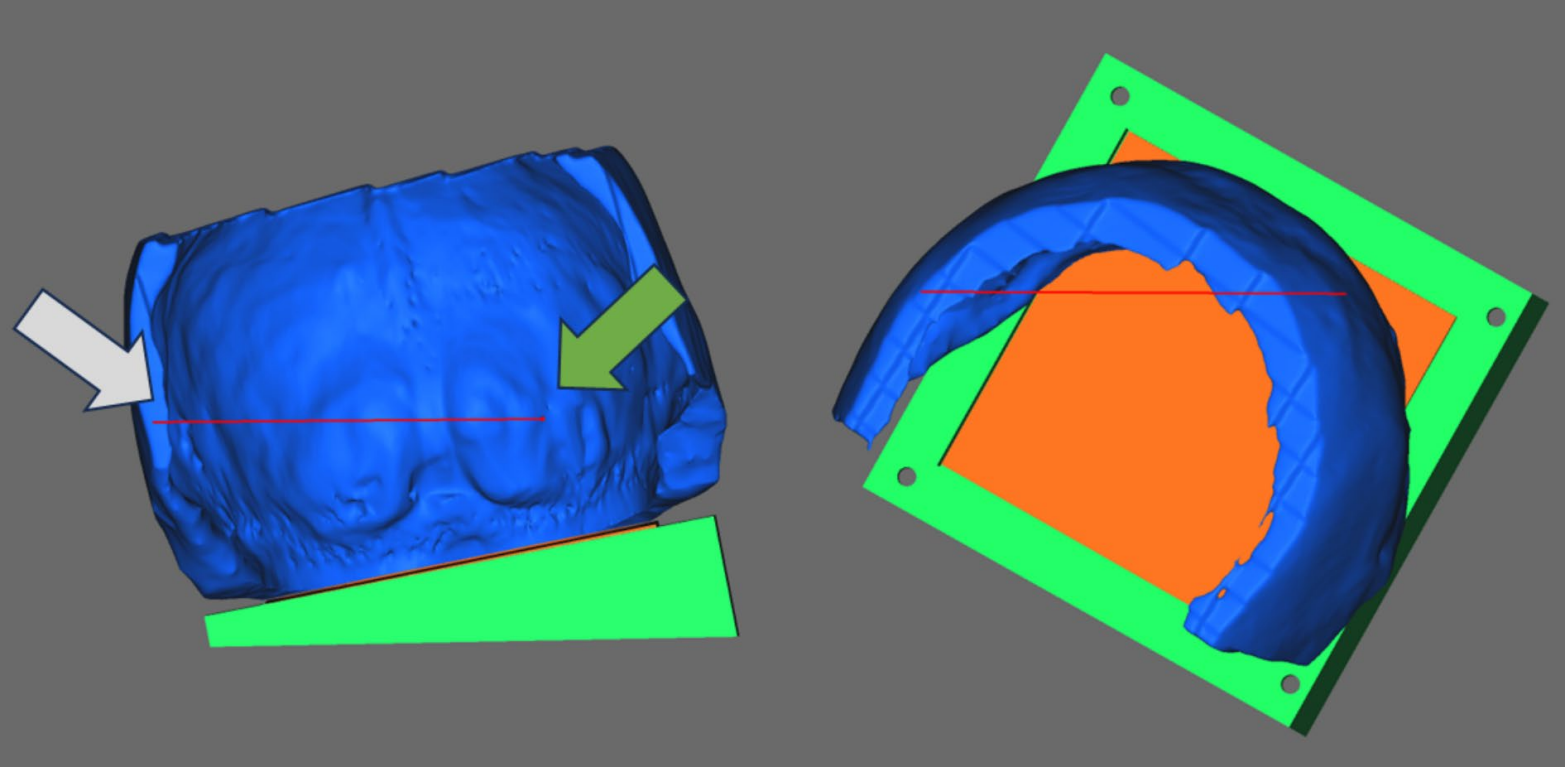
Representation of the head model, positioned for a shot on the customised plastic structure

According to the forensic pathologist’s findings, the questioned trajectory was from the right to the left, from the front to the back and from the top downwards. In order to facilitate the shooting session, a plastic structure has been built to fix the head model in a position that aligns the firing line (red line in Fig. 5) with the entry hole (grey arrow) and the location of the ricochet (green arrow) as shown in Fig. 5.

A cardboard box filled with Kevlar® was placed behind the model to catch any projectiles that might have completely perforated the head model.

#### Data analysis

For each shot, the following findings were reported: (a) a general description of the wound channel into the head model; (b) a measurement of the wound channel into the model performed by the mean of the CT; (c) a qualitative description of the entry wound; and (d) a qualitative description of the projectile deformation.

## Results

### Preliminary tests

**Fig. 6:**
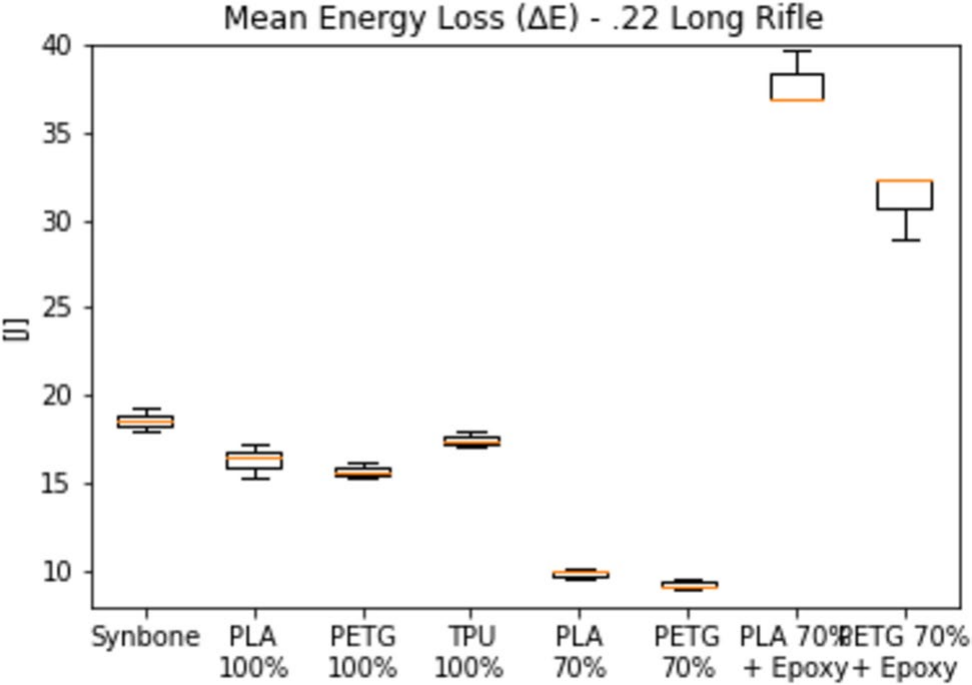
Energy loss of all tried materials under ballistic loading from a .22 Long Rifle bullet

Because no data could be found in the literature on 3D-printed bone simulants under ballistic loading, the complete list of results for the preliminary tests is presented. Figure 6 gives the energy loss of a 0.22 Long Rifle bullet, fired with a mean velocity of 363 ± 6 m/s through 6 mm thick samples. On the left of the graph are the results of a Synbone® sample of equal thickness, taken as a reference.

**Fig. 7:**
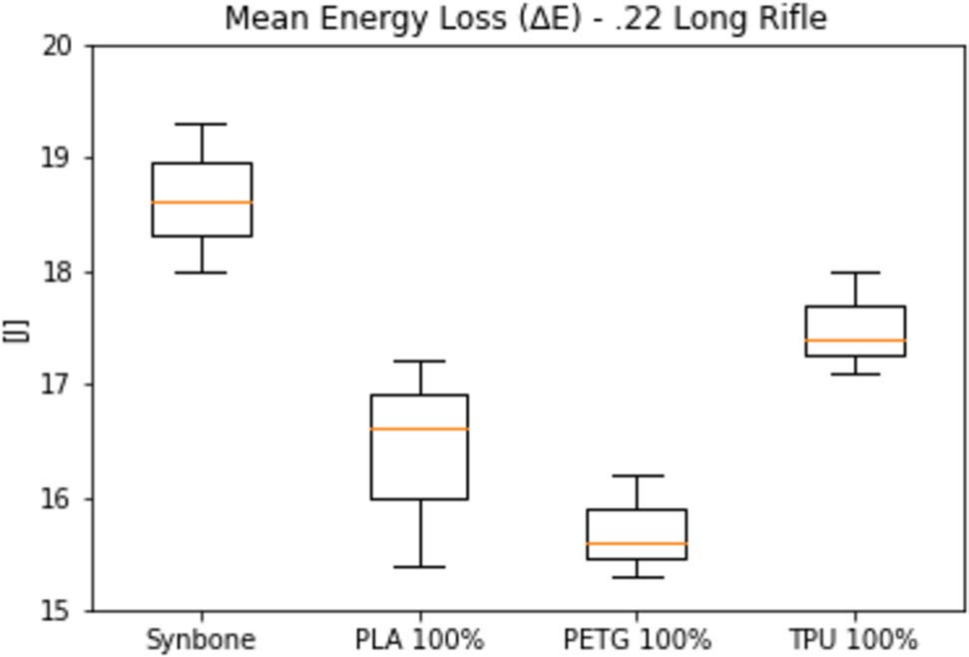
Energy loss of Synbone®, PLA 100%, PETG 100% and TPU 100% under ballistic loading from a .22 Long Rifle bullet

As observed, the energy loss in PLA and PETG samples with a 70% infill density was too low. The energy loss in samples of the same materials coated with epoxy resin was excessively high. Due to these significant discrepancies, both the 70% infill density samples and those treated with epoxy resin were excluded from further testing. To better appreciate the similarities between Synbone® and the PLA 100%, PETG 100% and TPU 100%, the following figure is focused only on these materials (Fig. 7).

**Fig. 8:**
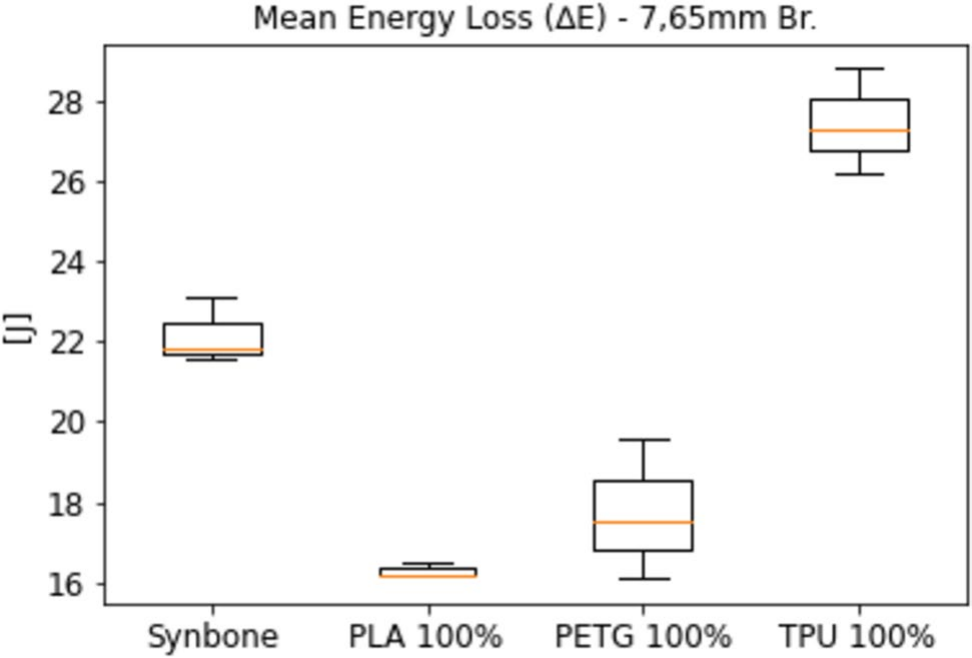
Energy loss of a selection of materials under ballistic loading from a 7.65 mm Browning FMJ bullet

Figure 8 gives the energy loss of a 7.65 mm Browning FMJ bullet, fired with a mean velocity of 270 ± 6 m/s through a selection of the samples from Fig. 6 and through Synbone® sample as a reference.

As shown in Fig. 8, the results for the PLA, PETG and TPU samples with a 100% infill density were close but not identical to those for Synbone®. Since Synbone® itself is only an approximation of human bone, this difference was not seen as problematic, and all three materials were considered as potential bone simulants for further tests.

The images of the entry and exit holes into the Synbone® plates as well the 3D-printed plates can be consulted in the appendix 8.1.

### Shooting tests on the 3D-printed head models

Three models were prepared with PLA 100% (named PLA 1, PLA 2 and PLA 3), three with PETG 100% (named PETG 1, PETG 2 and PETG 3) and three with TPU 100% (named TPU 1, TPU 2 and TPU 3). The shooting tests on the 3D-printed head models consisted of a total of eleven shots. The two BJT and MJT samples are named Powder and Resin respectively in the results. Shortly before each shot, the gelatine temperature in the head model was measured at a depth of approximately 4 cm. All temperature measurements were between 3.9 °C and 4.3 °C.

#### General description and wound channel length

Table 2 summarises the results observed for each shot against the eleven head models as well as the interaction type between the projectile and the occipital bone wall (second layer) and the wound track length. The images of the entry and exit holes into the bone simulants can be consulted in the appendix 8.2.

**Table 2:** General results, projectile/occipital bone wall interaction and wound track length observed for each head model

Model	General result	Second bone simulant layer in occipital left region	Wound track length in cm^a^
PLA 1	Retained inside the model between the two internal bone walls	-	4.5^b^
PLA 2	Head model completely perforated	Perforated	11.3
PLA 3	Head model completely perforated	Perforated	11.3
PETG 1	Retained by the gelatine outside the skull by the last gelatine layer	Perforated	11.1
PETG 2	Retained by the gelatine outside the skull by the last gelatine layer	Perforated	11.5
PETG 3	Head model completely perforated	Perforated	11.5
TPU 1	Ricochet against the occipital left bone	Mark of the ricochet on the bone simulant. No fracture	11.0 + 2.7
TPU 2	Retained by the gelatine outside the skull by the last gelatine layer	Perforated	11.4
TPU 3	Retained inside the model between the two internal bone walls. Impact with the occipital left bone	Fracture of the occipital left bone	11.7
Powder	Head model completely perforated	Perforated	11.8
Resin	Retained inside the model between the two internal bone walls. In contact with the occipital left bone	Small fracture (hole) at the point of impact	11.2

^a^The measurements were performed along the wound channel between the two internal bone walls as shown in Fig. 9 (temporal right and occipital left)

^b^The aiming point was slightly aside where the bone simulant was significantly thicker

**Fig. 9:**
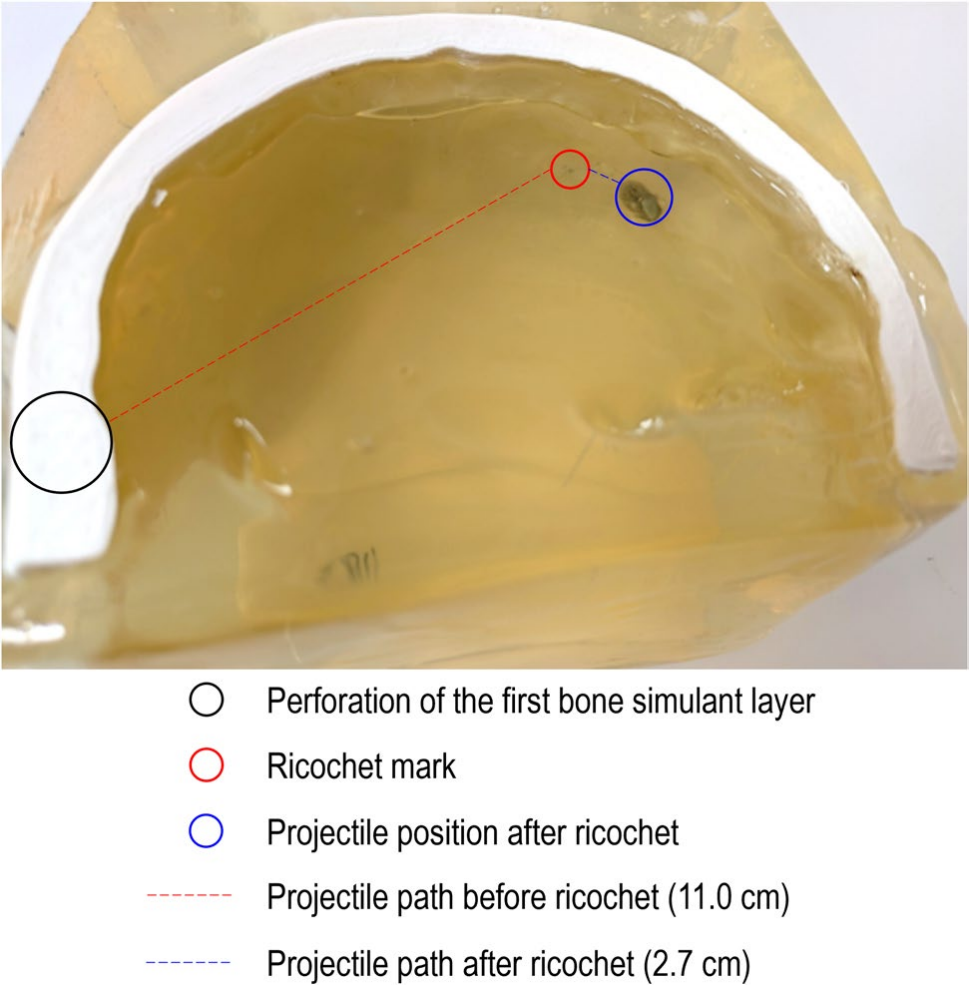
Shot #1 on the head model, prepared with a TPU bone simulant. The bullet’s path was close to that of the fatal shot

Shot #1 with TPU (TPU 1) has been the only shot where the observations in terms of wound characteristics were similar to the fatal head shot, with the exception of the length of the path after ricochet. The projectile path into the head model is shown in the following image (Fig. 9).

#### Bullet deformation

**Fig. 10:**
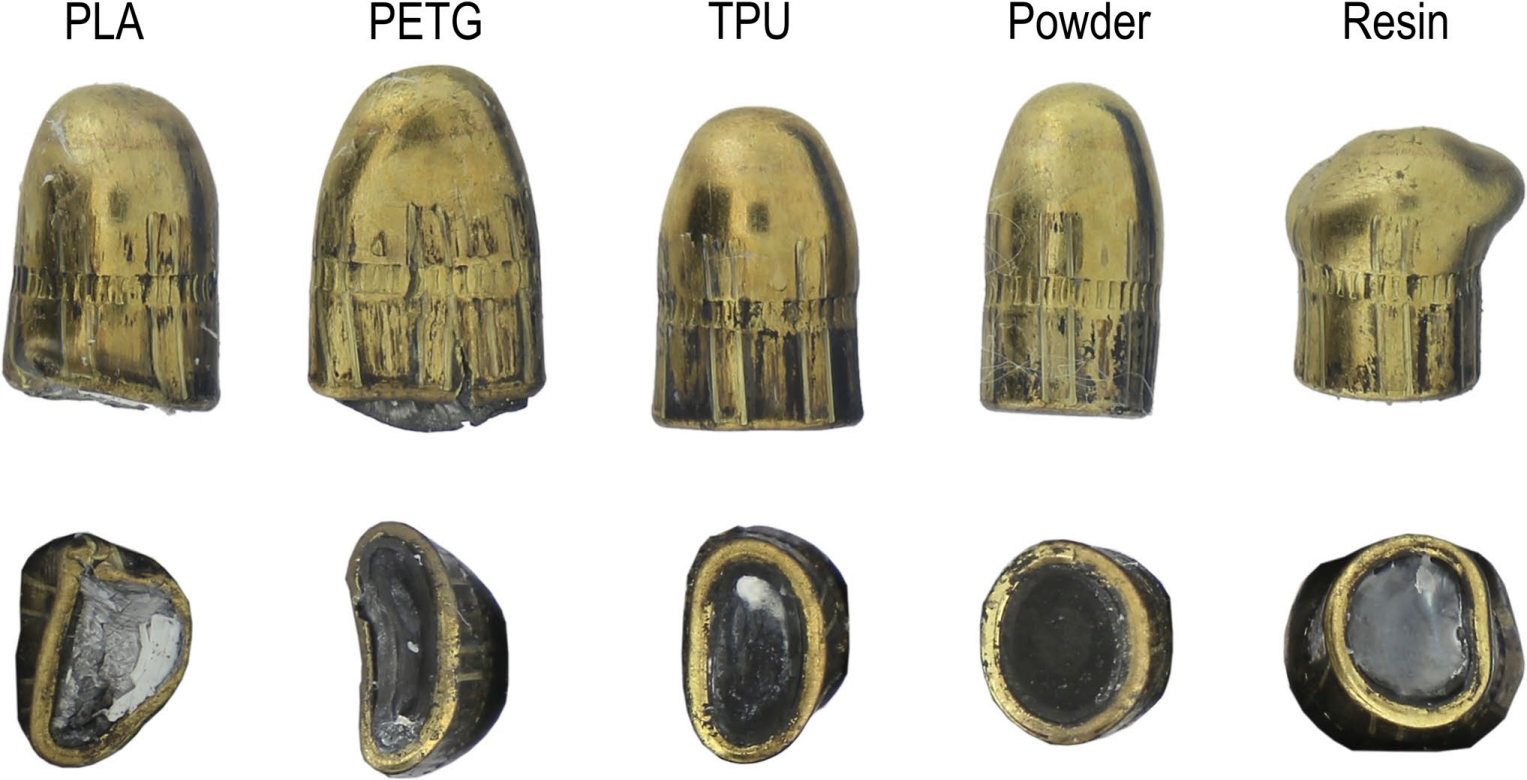
Bullet deformation on the various head models

The assessment of the deformation focused on the base, the body and the tip of the recovered bullets. The projectiles shot against the head models with PLA 100% and PETG 100% simulants showed a moderate deformation of the body, a flattened/deformed base as well as a rounded and less deformed tip (first and second projectiles from the left in Fig. 9). The moderate deformation can be explained by the perforation of two bones layers, as all but one bullet (TPU 1) fully perforated the models. The only projectile, which rebounded against the occipital wall of the TPU model (Fig. 9), showed mainly a flattening of its base (Fig. 10, in the middle). This kind of deformation is similar to the projectile deformation observed against Synbone® plates during the head models tests performed by Riva et al., but it is still less pronounced than the deformation observed on the questioned bullet of the fatal headshot reported in [1].

The projectile shot through the powder composite does not show any particular deformation; the absence of deformation indicates low impact resistance (fourth projectile from the left in Fig. 10). Finally, the projectile shot against the head model with a resin composite bone simulant shows an important deformation of the tip and a part of the body (fifth projectile from the left in Fig. 10). This kind of deformation is similar to the questioned projectiles deformation reported in [1].

## Discussion

### Considerations

To the minds of the authors, the procedure that was tested in this study has a lot of potential for future forensic casework. Although the procedure can still be improved, it was possible to replicate the shape and size of a victim's bone from PMCT data with a 3D printer. The results under ballistic loading of some 3D-printed materials were close to, but not identical to that of Synbone®. The fact that it was not identical to Synbone® is not alarming per se, because there is no definite proof that Synbone® itself always behaves identical to human bone under ballistic loading. By incorporating 3D-printed bone simulants in a head model, it was possible to partially replicate the outcome of a well-documented fatal headshot. Although most results were not similar to the head shot, the authors believe that the model can be improved after further experimentation. Using 3D-printed material gives the opportunity to adapt the shape and size of a bone simulant to the needs of a case at hand. By changing the printing parameters (e.g. infill density) it might also be possible to further individualize bone simulants, such as recreating bone with osteoporosis. After further development, 3D-printed bone materials might replace generic products as cost-effective and easy to use bone simulants for a large number of (forensic) applications.

### Limitations

The process still has limitations. The most relevant part of the bone in the PMCT data is damaged, while the simulant should replicate the bone in its undamaged condition prior to ballistic loading. This requires that data in the most relevant part of the bone (the part with the bullet defect) must be filled in. With small defects, e.g. caused by a 6.35 mm Browning bullet like in the current study, this is relatively easy. After shots with more energy deposition, which can cause fragmentation or even full comminution of bone, this will be more difficult. Another limitation is that the PMCT data only provides the overall bone thickness. Overall thickness might not be the only aspect that determines the strength of bone. The local thickness of cortical and trabecular bone is likely to be of influence as well, as will be the bone’s mineral density. These aspects are much harder (though not impossible) to assess from PMCT data.

### Further research

Further research should focus on more 3D-printable materials, with a slightly higher density and/or a slightly higher tensile strength. Materials such as carbon- or glass-fibre filled polymers or other types of resins seem promising. Other 3D printing approaches could also be tried. For example, it would be possible to 3D-print a skull mould in order to create bone simulants from liquid mixtures that solidify on drying.

## Conclusion

This study presents a novel concept, in reproducing case specific head models for ballistics tests based on PMCT data with 3D printing technology and easily available printing materials. Seeing the results of this study, the concept is promising, but the tested materials do not completely fulfil the requirements to simulate human bone. For this reason, more materials will have to be tested, and the results compared with well documented cases, existing bone simulants and/or donated human bone.

## Figures and Tables

**Fig. 11:**
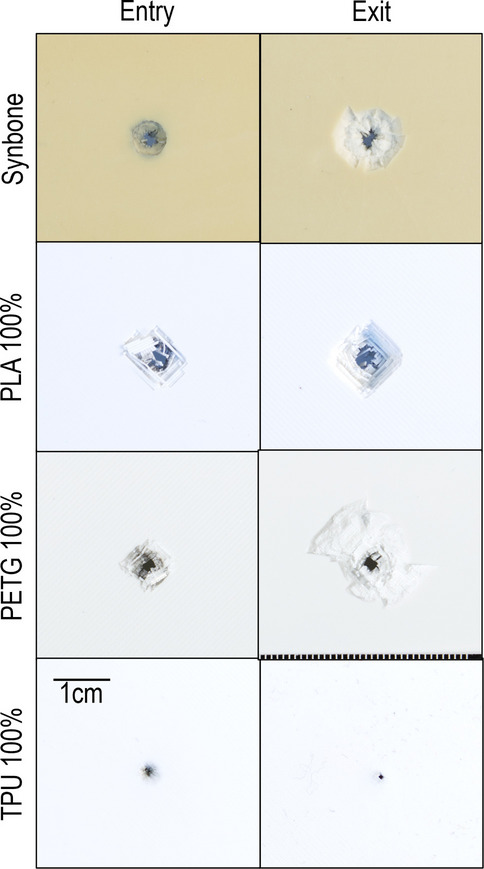
Entry holes (left column) and exit holes (right) left by .22LR LRN bullets shot against respectively (from the top to the bottom) Synbone, PLA 100%, PETG 100% and TPU 100% plates

**Fig. 12:**
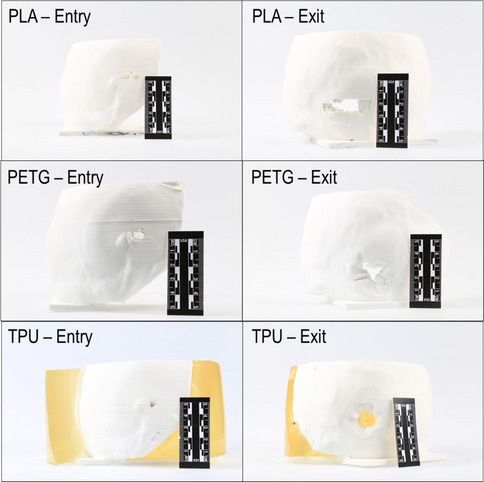
Entry holes (left column) and exit holes (right) left by 6.35 mm Browning FMJ bullets shot against the head models with skull simulants respectively printed with (from the top to the bottom) PLA, PETG and TPU

**Fig. 13:**
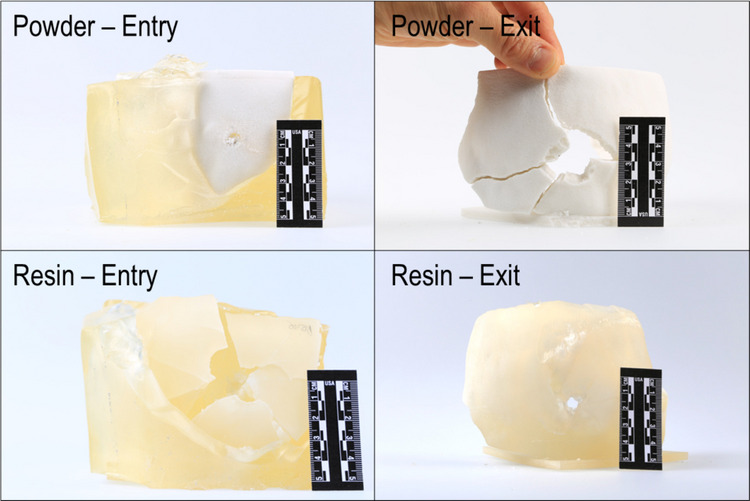
Entry holes (left column) and exit holes (right) left by 6.35 mm Browning FMJ bullets shot against the head models with skull simulants respectively printed with (from the top to the bottom) composite powder and resin

## Data Availability

Published data are available and can be requested by contacting the corresponding author.

## Appendix

### Qualitative aspects of the entry and exit holes on the perforated plates for the calibre 0.22LR

See Fig. 11

### Qualitative aspects of the entry and exit holes in the different head models with the calibre 6.35 mm Browning

Images showing the entry and exit hole in the different head model shot with the 6.35 mm Browning. The images have been done after removing the leather layer as well as most of the gelatine into the model.

See Figs. 12 and 13
